# A multi-scale small object detection framework for tomato leaf pest and disease detection in complex natural environments

**DOI:** 10.3389/fpls.2026.1923215

**Published:** 2026-09-11

**Authors:** Rui Gao, Shuang Ma, Meng Wang, Junyong Zhang, Jie Yang, Zhuoran Zhang, Shuai Yan, Dongrui Han

**Affiliations:** Institute of Agricultural Information and Economics, Shandong Academy of Agricultural Sciences, Jinan, Shandong, China

**Keywords:** deep learning, natural environments, pest and disease detection, small object, tomato leaf

## Abstract

**Introduction:**

This paper proposes an improved detection framework, PLD-YOLO, to address the challenges of small object scale, strong background interference, and loss of shallow feature information during feature extraction in pest and disease detection under complex natural environments for intelligent plant protection.

**Methods:**

The proposed framework enhances small object representation by introducing a high-resolution feature branch. It further improves contextual modeling capability and suppresses complex background noise through multi-scale convolutions and an adaptive feature mechanism. In addition, a dynamic small object weighting strategy is introduced to improve small object learning under scale imbalance conditions.

**Results:**

This study systematically evaluates the proposed method on the Tomato-Village dataset constructed under natural environments and compares it with representative two-stage object detection methods, YOLO series models, and transformer-based detection models. Ablation studies, cross-dataset validation, and robustness evaluations under different field conditions further demonstrate the effectiveness and robustness of the proposed approach. The experimental results show that PLD-YOLO achieves excellent performance in terms of precision, recall, mAP@50, and mAP@50–95 while maintaining high inference efficiency.

**Discussion:**

The proposed method provides a potential technical solution for intelligent plant protection and contributes to the advancement of precision agriculture.

## 1 Introduction

Tomato is one of the world’s most widely planted and consumed vegetable crops and is very vulnerable to pests and diseases during growth, which affects its yield and quality. According to statistics, pests and diseases may lead to a significant reduction in tomato yield in the absence of effective prevention and control measures (Canton, 2021). Common tomato pests and diseases include early blight, late blight and magnesium deficiency (Canton, 2021; Nagamani and Sarojadevi, 2022; Zhang et al., 2023). Therefore, timely and accurate detection of tomato pests and diseases is very important for agricultural production safety and for improving crop quality.

The traditional detection methods of tomato pests and diseases mainly rely on expert interpretation. They are labor-intensive, subjective, and inefficient, making it difficult to meet the needs of intelligent and large-scale development in modern agriculture (Li et al., 2022; Wang et al., 2025b). In recent years, deep learning techniques have made significant advances in various visual tasks, including image enhancement (Wang et al., 2023, 2024c, 2026), remote sensing visual question answering (Liu et al., 2026), and graph-based target classification and association (Li et al., 2025b, 2026). With the development of computer vision and deep learning technology, image-based pest detection methods have gradually become a research hotspot and have been widely used in agricultural production (Wspanialy and Moussa, 2020; Yuan et al., 2022). Existing deep learning pest and disease detection methods can simultaneously achieve the localization and classification of pests and diseases, which is far more practical than traditional methods.

At present, the commonly used object detection frameworks include Faster R-CNN (Ren et al., 2017), SSD (Liu et al., 2016), and YOLO (Redmon et al., 2016) series model architectures. Researchers have conducted extensive studies on tomato pests and diseases based on these models. This fully shows the great potential of deep learning technology in plant pest and disease detection. Mohanty et al. used a convolutional neural network to classify plant diseases on the PlantVillage dataset. They verified that CNN can achieve high-precision automatic extraction of pest features in a controlled environment (Mohanty et al., 2016). Wang and Liu added the deny module to the YOLO network and used the k-means algorithm to cluster the objects. This improved the accuracy of tomato detection to 96.41% (Wang and Liu, 2021). Jin et al. Fused multi-scale features by adding CBAM and weighted Bidirectional Feature Pyramid Network (BiFPN) to the Yolo network and developed an online disease detection platform (Jin et al., 2024). Bachri et al. used the collected pests and diseases dataset to create a detection model based on Fast R-CNN. This model can effectively detect various tomato pests and diseases (Bachri et al., 2024). The above methods provide a reference for tomato leaf pest and disease detection. However, most of the datasets used in these methods are obtained from laboratory environments. The backgrounds are simple and the influencing factors are controllable, so the practical accuracy cannot meet production needs.

Previous studies have shown that the detection performance of the model trained by the datasets in the laboratory environment will decline when it is migrated to the natural environment (Mohanty et al., 2016). This shows that complex backgrounds and varying lighting conditions have important effects on pest and disease detection. In real natural environments, tomato pests and diseases are affected by multiple factors, including their types and development stages, complex backgrounds, and varying lighting conditions (Ferentinos, 2018). These factors result in dense distribution, scale diversity, and blurred boundaries of tomato pests and diseases. In addition, small objects often suffer from insufficient feature representation during detection (Zhu et al., 2019), while blurred boundaries further increase the difficulty of localization. Therefore, during training the model is prone to three main problems. These are loss of small object features, difficulty distinguishing adjacent pests and diseases and inaccurate localization. At the same time, the complex natural environment further increases false and missed detections.

To solve the above problems, this study trains on natural environment datasets and improves multi-scale feature expression and training strategy based on YOLOv11-l. It constructs an improved tomato leaf diseases and pest detection model, PLD-YOLO. The main contributions of the dissertation are as follows:

1. The high-resolution P_2_ feature layer is introduced into the original three detection heads of YOLOv11-l, forming a ${\text{P}}_{2}-{\text{P}}_{5\;}$ multi-scale detection framework. This design mitigates the loss of shallow feature information during downsampling, enhances the representation capability of low-level features, and improves detection performance for densely distributed small objects and multi-scale pests and diseases.
2. A Lightweight Local Context Feature Enhancement (LCFE) module is proposed. It enhances shallow feature representation through multi-scale convolution and an adaptive feature fusion mechanism, enabling more effective integration of contextual information and improving discriminability in complex backgrounds, especially for adjacent lesions and blurred boundary regions.
3. A Dynamic Small Object Reweighted Loss (DSOR-Loss) is designed. It adaptively assigns higher weights to small objects based on their distribution within each batch, alleviating scale imbalance during training and improving performance in small object detection.

## 2 Related work

This paper summarizes the evolution of object detection and pest detection. In object detection, the field has developed rapidly due to advances in artificial intelligence and deep learning technology. At present, the algorithms for object detection can be divided into two categories. One is a two-stage detector represented by R-CNN (Girshick et al., 2014). They generate several candidate boxes according to the input picture and classifies the candidate boxes using a convolutional neural network. The other is the single-stage detector represented by SSD and YOLO algorithm. They regard detection as regression problem analysis and do not need to generate candidate boxes. The two-stage detector has high detection accuracy. However, its complex network structure and slow computational speed make it unsuitable for real-time detection. In contrast, the single-stage detector has the advantages of simple structure and fast reasoning speed. In recent years, with the continuous improvement of YOLO series models, their end-to-end training and multi-scale feature fusion mechanisms have been widely used in small object detection (Lai et al., 2023; Wu et al., 2024; Wang et al., 2025a).

The YOLOv1 model achieves end-to-end real-time detection by referring to the GoogLeNet classification network structure (Redmon et al., 2016). It formulates the object detection task as a regression problem. However, its detection ability on dense objects is poor and the accuracy is insufficient. To address this problem, Redmon introduced YOLOv2 in 2016 (Redmon and Farhadi, 2017). Compared with YOLOv1, YOLOv2 adds Batch Normalization (BN) (Ioffe and Szegedy, 2015) to each convolutional layer to accelerate the convergence of the neural network. Later, YOLOv3 model was proposed to predict different feature layers by introducing multi-scale detection mechanism (Redmon and Farhadi, 2018). This improves the detection ability of different size objects to a certain extent. However, its use of fine-grained features of small objects is still insufficient. Subsequently, the proposed YOLOv5 model improves the feature fusion ability by introducing the Cross Stage Partial Network (CSPNet) structure and the Path Aggregation Network (PANet). It achieves a good balance between accuracy and speed (Jocher et al., 2022). However, the anchor mechanism of YOLOv5 is sensitive to the change of object scale, and there is still the problem of inaccurate localization in small object detection. YOLOv8 optimizes the network structure and loss function while using an anchor-free detection mechanism. This further improves the model’s generalization ability (Yaseen, 2024).

The latest version of YOLO introduces several architectural improvements for small object detection. YOLOv9 improves information flow in deep networks by introducing Programmable Gradient Information (PGI) and the Generalized Efficient Layer Aggregation Network (GELAN) (Wang et al., 2024b). YOLOv10 introduces a consistent double allocation mechanism to enable NMS-free training and reduce inference delay (Wang et al., 2024a). YOLOv11 uses a Cross Phase Partial and Spatial Attention (C2PSA) module and an optimized anchor-free detector head. Among them, the C2PSA module combines cross phase partial connections with a spatial attention mechanism. Therefore, the model can effectively extract feature information in complex backgrounds while maintaining inference speed. The optimized anchor-free detector head reduces excessive dependence on prior anchors and improves the model’s adaptability to multi-scale objects (Khanam and Hussain, 2024). Therefore, YOLOv11 achieves a good balance between speed and accuracy while being stable in complex environments and small object detection. Although newer YOLO series models have been proposed (Saif et al., 2026; Sapkota et al., 2026; Yurdakul et al., 2026), the structure of YOLOv11 is relatively mature and widely used.

In pest and disease detection, early methods mainly relied on traditional image processing to determine the color, texture, and shape of disease spots through expert interpretation. Machine learning methods were then used to establish relationships to classify and recognize pests and diseases. Hussein and Abbas used support vector machine (SVM) to classify rice pests and diseases. They first selected a group of pest samples to build a knowledge base and combined them with texture features such as the Gray Level Co-occurrence Matrix (GLCM) for classification (Hussein and Abbas, 2019). These methods can achieve certain results under specific conditions. However, the feature representation is insufficient and cannot fully capture the diversity and uncertainty of disease spots. At the same time, it is difficult to meet the needs of practical production and application because of the interference of complex backgrounds and varying lighting on the model in the natural environment.

With the development of deep learning technology, crop pest and disease detection methods based on Convolutional Neural Networks (CNNs) has gradually become a research hotspot. Kulkarni and Shastri used a CNN to classify and recognize rice pests and diseases. They verified that CNN could effectively extract the feature information of pests and diseases under certain conditions (Kulkarni and Shastri, 2024). However, the above methods only consider pests and diseases as a classification problem. These methods focus solely on the presence of pests and diseases and do not address their location (Chen et al., 2024). In real natural environments, crop pests and diseases often occupy only a small part of the image (Zhang et al., 2025b). The modeling approach of classification methods easily ignores regional information and therefore has limited ability to perceive small pests and diseases. As a result, the detection performance of crop pests and diseases in real-world scenarios is also limited. To address this problem, researchers introduced object detection into plant pest and disease recognition. This achieved a leap from simple classification to precise localization.

YOLO series models are the most representative algorithms in object detection. With their continuous development, researchers have focused on creating practical pest detection algorithms. Many improvements have been proposed based on YOLO networks, effectively enhancing the efficiency and accuracy of pest detection. Ji et al. added a Soft Complete Intersection over Union (Soft-CIoU) loss function to YOLOv4. They combined it with multi-scale context information to address the detection difficulties caused by low resolution small objects (Ji et al., 2023). At the same time, Aldakheel et al. used YOLOv4, DenseNet, AlexNet, and other models to analyze images of 14 plant diseases and pests. The results showed that YOLOv4 achieved the best performance on the dataset (Aldakheel et al., 2024). Soeb et al. achieved high-precision detection of tea plant diseases in natural environments using the YOLOv7 model and data augmentation methods (Soeb et al., 2023). In terms of attention mechanisms, Li et al. built a rice disease detection model by integrating the attention mechanism of YOLOv5 with the lightweight design of GsConv. The model achieved high-precision detection of rice diseases (Li et al., 2025a). Zhang et al. improved the YOLOv8 model by using the MobileNetV3 backbone and multiple attention mechanisms. This enhanced the accuracy and efficiency of rice pest detection (Zhang et al., 2025a).

Although these improvements have achieved good performance in plant pest and disease detection, challenges still remain in tomato leaf pests and diseases detection. Firstly, tomato leaf pests and diseases exhibit significant scale variation. Small objects are prone to information loss during deep feature propagation, resulting in insufficient feature representation. Secondly, different types of pests and diseases show variations in texture and scale across different growth stages, which further increases the difficulty of detection. In addition, complex backgrounds, illumination changes, and leaf occlusions in natural environments lead to missed and false detections. Finally, existing methods still have limitations in feature representation and multi-scale feature fusion, making it difficult to meet the performance requirements under complex natural environments. Therefore, improving small object feature representation and enhancing discrimination ability in complex backgrounds remain key challenges to be addressed.

## 3 Methodology

### 3.1 Overall architecture

**Figure 1:**
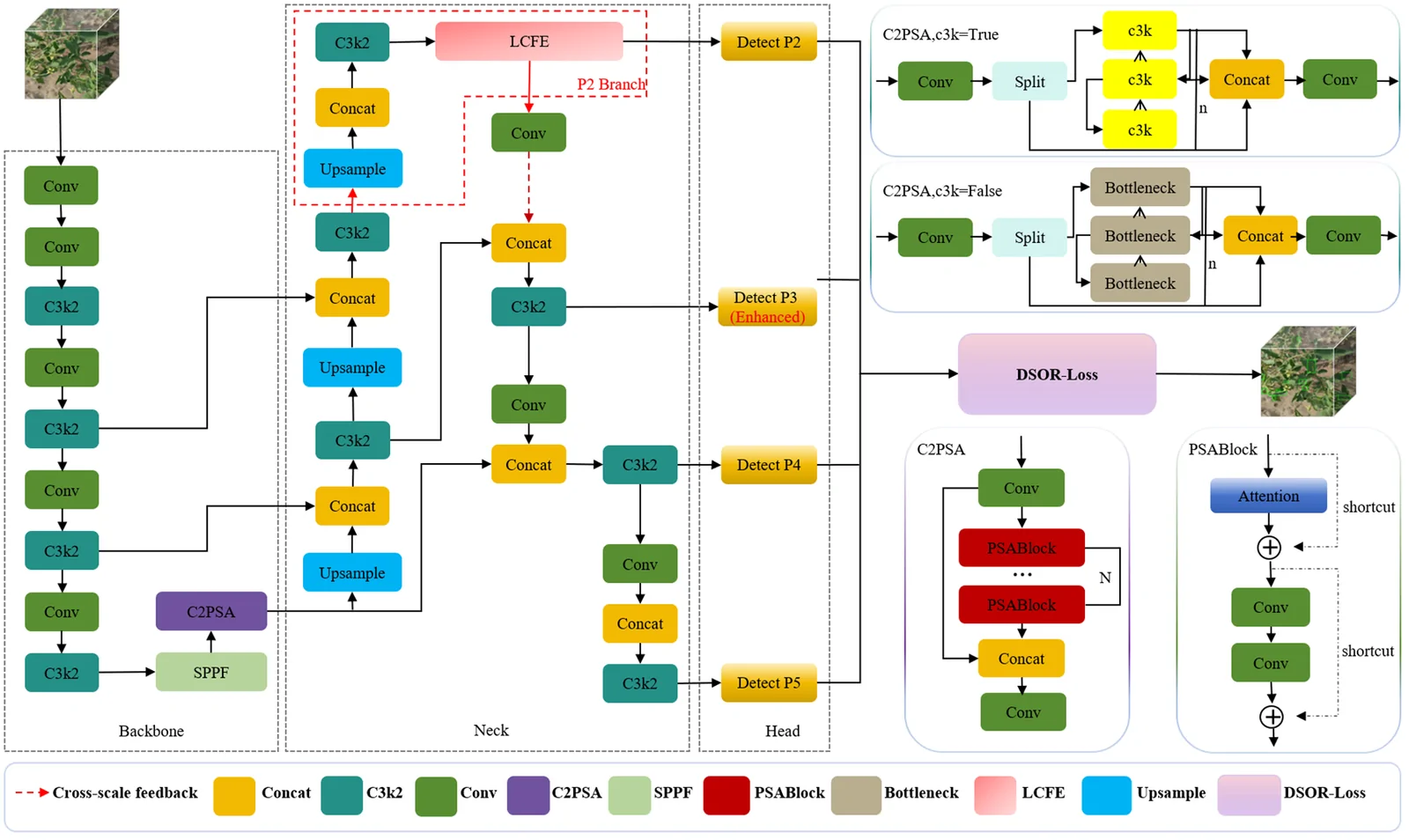
The proposed YOLOv11-l architecture with P_2_ detection layer, LCFE module, and DSOR-Loss. The entire red dashed box represents the P_2_ high-resolution branch, which includes the LCFE module. Additionally, a backflow mechanism is applied to propagate enhanced features from P_2_ back to P_3_. The DSOR-Loss is introduced during training to adaptively emphasize small objects.

In this study, YOLOv11-l was selected as the baseline model, and the improved PLD-YOLO model was proposed. YOLOv11-l incorporates advanced feature representation and detection mechanisms. Its C2PSA module and anchor-free detection mechanism enhance learning in complex backgrounds and for multi-scale objects. The model shows strong robustness and stability in small object detection. Although YOLOv11-l requires higher computational demands than lightweight models, it has strong feature representation capability. This is especially true for detecting small object leaf pests and diseases. Firstly, the P_2_ high-resolution detection branch is introduced to reduce the loss of small object features. It is fused with other branches, and the detection accuracy of small objects is enhanced by improving the feature resolution. Secondly, the LCFE is introduced to enhance the expression ability of local key feature information. This allows local texture information and high-level semantic information to be better integrated. At the same time, by constructing the cross-scale feature propagation path from P_2_ to P_3_, reverse supplementation from shallow information to high-level semantic information is realized. This improves the discrimination ability of low-level features. Finally, DSOR-Loss is used to replace the bounding box loss function. The dynamic weighting of small objects is adaptively adjusted to avoid the neglect of small object features caused by excessive attention to large objects during training, and to reduce the omission of small objects. The structure of PLD-YOLO is shown in **Figure 1**.

### 3.2 High-resolution P_2_ detection layer

The object detection network obtains richer semantic information by continuously deepening the convolution layer. However, it is easy to cause the details to be blurred, which will lead to the decline of detection accuracy. In the detection of tomato pests and diseases, the disease spots usually have the characteristics of dense distribution, diverse scales and irregular distribution. In particular, the low proportion of small object lesions pixels makes it difficult to be effectively expressed in deep features. With the deepening of the number of network layers, the resolution of the feature maps continues to decrease. This results in the loss or weakening of spatial details of small objects and increases the likelihood of missed or false detections. Therefore, the network directly outputs and fuses low-level feature information. This preserves its high-resolution and rich feature details. As a result, the network’s performance in small object detection is improved. Based on the original ${\text{P}}_{3}-{\text{P}}_{5}$ detection layers of YOLOv11-l, we built a new P_2_ high-resolution detection branch in the neck stage. This branch was expanded to form a ${\text{P}}_{2}-{\text{P}}_{5}$ multi-scale detection layer. At the same time, the reverse fine-grained information supplementation path from P_2_ to P_3_ is introduced to enhance the detail expression of middle-level features. First, the feature map $F_{3}$ corresponding to layer P_3_ is upsampled and fused with the feature map $F_{2}$ corresponding to layer P_2_ as shown in Equation (1).

(1) $${\text{F}}_{2}^{\prime }=\text{Concat}(\text{Upsample}({\text{F}}_{3}),{\text{F}}_{2})$$

here, $F_{2}$ and $F_{3}$ denote the feature maps from the P_2_ and P_3_ layers, respectively, and $F_{2}^{'}$ represents the initial fused feature map obtained by concatenating these two feature maps.

(2) $${\hat{F}}_{2}=H({\text{F}}_{2}^{\prime })$$

here, ${\hat{F}}_{2}$ denotes the reconstructed and enhanced feature map of the P_2_ layer and H(·) refers to the C3 feature reconstruction module based on the CSP idea as shown in Equation (2). The module divides the input feature into two paths. One path is used for convolution and bottleneck feature extraction. The other path retains the original information. Finally, the information is fused by feature stitching. This enhances feature expression and improves gradient propagation efficiency.

Because ${\hat{F}}_{2}$ has higher resolution, it can directly retain the details of small objects, thereby improving the detection sensitivity for small pest and disease objects. In addition, during the training process, the enhanced feature ${\hat{F}}_{2}$ generated from the P_2_ layer is re-fused with feature map $F_{3}$ from the P_3_ layer to supplement and enrich the semantic information of P_3_. This process is referred to as the backflow mechanism as shown in Equation (3).

(3) $${\text{F}}_{3}^{\prime }=\text{γ}({\hat{\text{F}}}_{2},{\text{F}}_{3})$$

here, ${\text{F}}_{3}^{\prime }$ represents the enhanced P_3_ feature map obtained through cross-scale fusion and γ(·) represents the cross-scale fusion function. The alignment and fusion of feature maps from different levels are achieved through downsampling and concatenation. Finally, the multi-scale features participate in detection as shown in Equation (4).

(4) $$\text{Y}=D({\hat{F}}_{2},{\text{F}}_{3}^{\prime },{\text{F}}_{4},{\text{F}}_{5})$$

here, Y denotes the final multi-scale feature representation used for detection, and D(·) represents the detection head.

The model introduces the P_2_ branch and the backflow mechanism. This not only improves the performance of small object detection but also enables effective interaction of cross-scale feature information.

### 3.3 LCFE: multi-scale context enhancement for tomato leaf diseases detection

**Figure 2:**
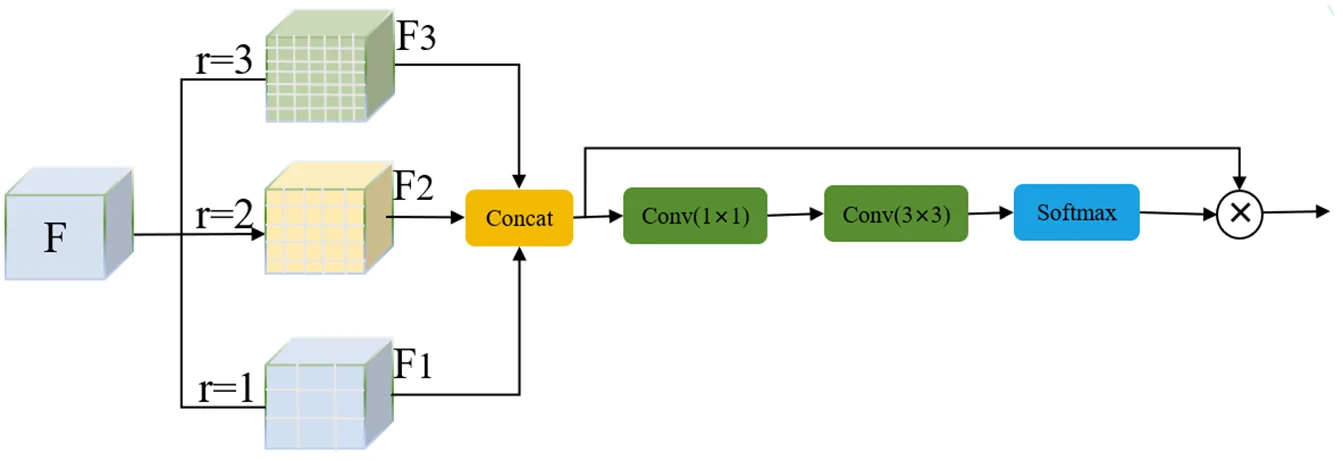
Architecture of LCFE.

In convolutional neural networks, the receptive field is predetermined. This limits the network’s ability to model information at different scales. In tomato leaf pest and disease detection, the features of pests and diseases often show local aggregation, diverse scales, fuzzy boundaries, and occasional occlusion. These features result in limited information. Therefore, it is not enough to fully extract the lesion information only by adding high-resolution features. So, this paper introduces the LCFE (Jiang et al., 2024). The network architecture is shown in **Figure 2**. Through multi-scale convolution and adaptive feature fusion, the context between the lesion and the surrounding environment is enhanced. As shown in the **Figure 2**, the input feature map F is processed, and multi-scale features are obtained through convolution operations with expansion rates *r=1,2,3*, respectively as shown in Equation (5).

(5) $${\text{F}}_{\text{i}}={\text{DilatedConv}}_{\text{r}=\text{i}}(\text{F}),\text{i}\in \{1,2,3\}$$

here, F denotes the input feature map, ${\text{F}}_{\text{i}}$ represents the feature map generated with the expansion rate r=i,where i denotes the scale index. Then, the feature maps at three scales are concatenated to obtain the fused features as shown in Equation (6).

(6) $${\text{F}}_{\text{cat}}=\text{Concat}({\text{F}}_{1},{\text{F}}_{2},{\text{F}}_{3})$$

here, ${\text{F}}_{\text{cat}}$ represents the concatenated feature map obtained from multi-scale features. Adaptive feature fusion is used to allocate weights for features at different scales. First, a 1×1 convolution with three output channels is applied to reduce the feature dimension. Then, a 3×3 convolution is used to enhance spatial modeling. Finally, the softmax activation function is applied to normalize the fused features and obtain the weight coefficients for each scale as shown in Equation (7).

(7) $$\alpha =\text{Softmax}(\text{Conv}(\text{Conv}({\text{F}}_{\text{cat}})))$$

here, α denotes the adaptive weight coefficients for different-scale features. Finally, the multi-scale features are weighted and fused to obtain the output feature $F_{c}$ as shown in Equation (8).

(8) $${\text{F}}_{\text{c}}=\alpha \cdot {\text{F}}_{\text{cat}}$$

here, ${\text{F}}_{\text{c}}\;$represents the output feature map after adaptive multi-scale fusion.

Through the above operations, additional context information is aggregated from multiple scales. This can enrich the feature representation and improve detection performance with minimal additional computation.

### 3.4 DSOR-Loss: a dynamic small object optimization strategy

In YOLOv11-l, the overall loss consists of the bounding box regression loss, the classification loss, and the distribution focal loss as shown in Equation (9).

(9) $$L=L_{\text{box}}+L_{\text{cls}}+L_{\text{dfl}}$$

here, $\;L_{\text{box}}$ represents the bounding box regression loss based on the Complete Intersection over Union (CIoU) metric. CIoU considers the overlap area, the distance between the centers of predicted and ground-truth boxes, and the difference in aspect ratio, thereby improving regression stability and convergence speed. $L_{\text{cls}}$ denotes the classification loss used to determine the object category. $L_{\text{dfl}}$ represents the distribution focal loss. By discretizing the bounding box coordinates into a probability distribution, the localization accuracy is optimized, especially in complex backgrounds and small object detection.

It should be noted that although each loss term is usually assigned a weight coefficient during training to balance the importance of different tasks, the weight is omitted in the Equation 9 here to emphasize the structural relationship of each component.

Due to the small-scale and uneven distribution of tomato leaf disease spots, the traditional regression loss is easily dominated by large objects during training, which results in insufficient learning of small objects. Therefore, this paper introduces a dynamic small object optimization strategy on $L_{\text{box}}$, while $L_{\text{cls}}$ and $L_{\text{dfl}}$ are left unchanged.

First, the scale is defined based on the normalized area of the object box as shown in Equation (10).

(10) $$A_{i}=\frac{w_{i}}{W}\times \frac{h_{i}}{H}$$

here, $A_{i}$ represents the normalized area ratio of the object box. $w_{i}$ and $h_{i}$ denote the width and height of the object box, respectively, and *W* and *H* denote the dimensions of the input image. In each batch during model training, the normalized scale distribution of all bounding boxes in the current batch is automatically computed. Based on this scale distribution, the top 15% of boxes are regarded as small objects. The small objects threshold $A_{\text{th}}$ for the current batch is dynamically determined. This threshold is then used to classify the objects into small and conventional objects.

On this basis, the regression loss corresponding to the small objects is dynamically weighted as shown in Equation (11).

(11) $$\omega_{i}=\{\begin{array}{rr} \lambda \cdot (1+r_{s}), & A_{i}<A_{\text{th}}\\ 1,\;\;A_{i}\geq A_{\text{th}} \end{array}$$

here, $\omega_{i}$ denotes the dynamic adaptive weight and λ is the amplification factor for small objects. In this experiment, λ=2.8. $r_{s}$ is the proportion of small objects in the current batch and $A_{\text{th}}$ is the threshold of small objects in the current batch. The weighted bounding box regression loss after dynamic adaptive weighting is calculated as shown in Equation (12).

(12) $$L_{box}^{'}=\sum_{i=1}^{N}\omega_{i}\cdot L_{box}^{i}$$

The total training loss is defined as shown in Equation (13).

(13) $$L_{\text{total}}=L_{\text{box}}^{'}+L_{\text{cls}}+L_{\text{dfl}}$$

here, $\;L_{\text{box}}^{'}$ denotes the weighted bounding box regression loss after dynamic adaptive weighting and $L_{\text{total}}$ denotes the total training loss.

By introducing DSOR-Loss, the model can adaptively enhance the perception of small pest and disease objects under different image conditions during training. It effectively reduces the missing detection rate of small objects and improves the overall detection accuracy.

## 4 Experimental results and analysis

### 4.1 Dataset introduction and experimental setup

#### 4.1.1 Dataset

In recent years, the PlantVillage dataset has been commonly used in pest detection research (Hughes and Salathe, 2016). This dataset contains samples of various plant pests and diseases, and is characterized by a large-scale and standardized labeling. However, most datasets are collected in laboratories or controlled environments, with a single background and limited interference. This limits the generalization ability of models trained on them in real-world field environments. In order to better reflect real agricultural scenarios, the Tomato-Village dataset published in 2023 is selected for this experiment.

**Figure 3:**
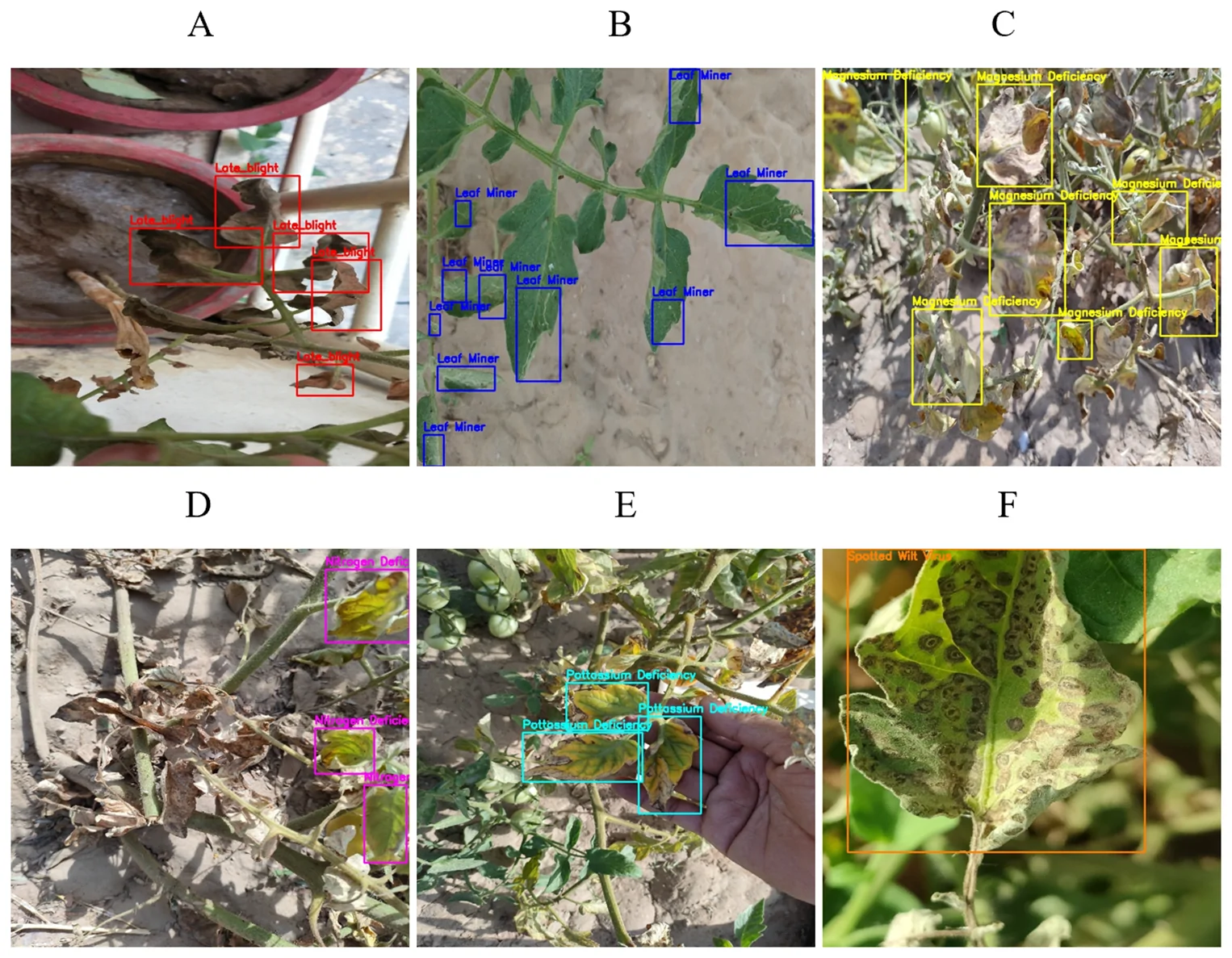
Plant disease types in the dataset. Each image highlights the disease features with a green box. The disease types are as follows: **(A)** late blight; **(B)** leaf miner; **(C)** magnesium deficiency; **(D)** nitrogen deficiency; **(E)** potassium deficiency; **(F)** spotted wilt virus.

This study uses the object detection subset of the Tomato-Village dataset, which contains 14,358 tomato leaf images (Gehlot et al., 2023). The images were collected from farmland in Rajasthan, India. They include tomato leaves in natural growth conditions and real pest images provided by farmers. The dataset covers tomato leaves at different growth stages under varying lighting and natural environmental conditions. The dataset contains six types of diseases: late blight, leaf miner, magnesium deficiency, nitrogen deficiency, potassium deficiency, and spotted wilt virus. The categories of tomato leaf pests and diseases are shown in **Figure 3**.

**Figure 4:**
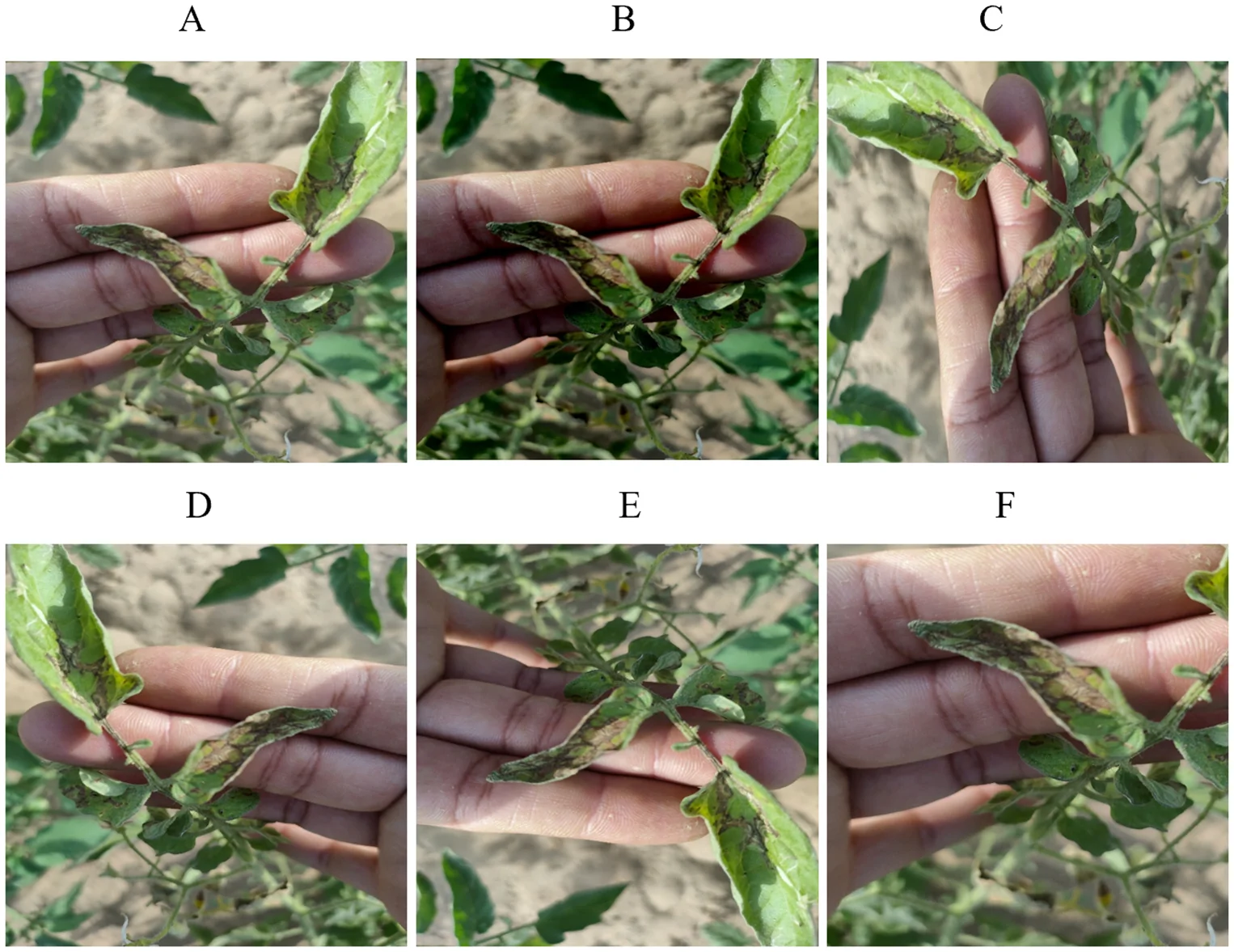
Data augmentation methods: **(A)** Original image; **(B)** Brightness adjustment; **(C–E)** Rotation; **(F)** Zoom.

As shown in **Figure 4**, to improve the generalization ability of the model in complex environments, data augmentation operations such as random flipping, scaling, and lighting adjustment are applied to the dataset. At the same time, considering differences in sample quality in the original dataset, low-quality samples such as those without annotations were removed. Finally, 10,344 images were selected as the experimental dataset. The dataset is divided into training set, validation set and test set according to the ratio of 7:2:1.

The experiment was conducted on a Windows system. Python version 3.9.21 was used with an Anaconda environment configured for CUDA 11.7 and PyTorch 1.13.1. The experiments were performed on an NVIDIA RTX A4000 GPU with cuDNN enabled. The model was trained with an input image size of 768 × 768 pixels, 300 epochs, a batch size of 8 images, and an initial learning rate of 0.001. Training was initialized using pre-trained weights. All baseline models used the same training parameters and data augmentation strategies to ensure a fair comparison. For computational efficiency evaluation, the inference speed (FPS) and GFLOPs of all models were measured under the same hardware environment with a batch size of 1.

Under identical training settings, we conducted additional experiments on the input image resolution. The results show that when the input size is reduced from 768×768 to 640×640, the model’s mAP@50–95 decreases by 1.58%. When the input size is increased from 768×768 to 896×896, the accuracy improves only slightly, but the inference speed is significantly slower. Therefore, the input size in this study is set to 768×768 pixels.

#### 4.1.2 Evaluation metrics

We use commonly adopted evaluation metrics, including recognition accuracy, localization accuracy, and detection speed.

Accuracy represents the proportion of correctly detected bounding boxes to all ground truth boxes. Recall represents the proportion of correctly detected bounding boxes to all detected boxes. These can be expressed by Equations 14 and 15:

(14) $$Precision=\frac{\text{TP}}{\text{TP}+\text{FP}}$$

(15) $$Recall=\frac{\text{TP}}{\text{TP}+\text{FN}}$$

here, TP indicates that a detected tomato leaf pest or disease is correctly identified, FP indicates that a detected tomato leaf pest or disease is incorrect, and FN indicates that a tomato leaf pest or disease is missed.

In addition, the Intersection over Union (IoU) threshold is used to determine the upper parameters. IoU measures the overlap between the predicted bounding box and the ground truth box. Its value ranges from 0 to 1, with higher values indicating greater localization accuracy.

By plotting precision on the x-axis and recall on the y-axis, the resulting curve is called the precision–recall curve. The area under this curve is referred to as average precision (AP), which serves as a comprehensive metric for evaluating the object detection performance of the model, as shown in Equation 16.

(16) $$\text{AP}=\int_{0}^{1}P(R)\text{d}R$$

here, P(R) represents the precision corresponding to the recall *R*.

For multi-class objects, the average precision of each class can be calculated, which is referred to as mean average precision (mAP). mAP measures whether the predicted bounding boxes accurately detect all categories and positions, reflecting the robustness of the algorithm across all classes in the dataset, as shown in Equation 17.

(17) $$\text{mAP}=\frac{\sum_{n=1}^{N}\text{AP}(n)}{N}$$

here, *n* denotes the class index, and *N* denotes the total number of classes.

In addition, we evaluated the mAP values at different IoU thresholds. When the IoU threshold was 0.5, the AP for each class was calculated. When the IoU ranged from 0.5 to 0.95 in steps of 0.05, the mean AP over multiple thresholds reflects the model’s localization accuracy under more stringent criteria. To assess the model’s computational complexity and inference efficiency, we also measured the floating-point operations (GFLOPs) and inference speed (FPS) of different models (Cheng et al., 2024).

### 4.2 Comparative experiments

In this study, several object detection models, including Faster R-CNN (Ren et al., 2017), RT-DETR (Kong et al., 2024), RetinaNet (Wang et al., 2019), YOLOv5 (Jocher et al., 2022), YOLOv8 (Yaseen, 2024), YOLOv9 (Wang et al., 2024b), YOLOv10 (Wang et al., 2024a), YOLOv12 (Yurdakul et al., 2026), YOLOv11 (Khanam and Hussain, 2024) and the proposed PLD-YOLO model, were compared to evaluate the effectiveness of the proposed method in tomato pest and disease detection. To ensure fairness and comparability of the experimental results, all models were trained using the same dataset partition and training strategy. Their performance was then evaluated on the test set.

**Table 1** presents the comparison results between the proposed PLD-YOLO and object detection models on the validation set. As shown in **Table 1**, the precision, recall, mAP@50 and mAP@50–95 of PLD-YOLO are 97.75%, 96.77%, 98.58% and 84.08%, respectively. Compared with other object detection models, PLD-YOLO shows good detection performance. Compared with YOLOv11-l, PLD-YOLO improves precision, recall, mAP@50 and mAP@50–95 by 3.35%, 4.51%, 2.50% and 2.47%, respectively, indicating that the model demonstrates clear advantages in detection accuracy and recall.

**Table 1:** Performance comparison of different models on tomato pest and disease detection.

Network model	Precision/%	Recall/%	mAP@50/%	mAP@50-95/%	GFLOPs	FPS/s
Faster R-CNN	85.48	75.50	64.48	40.42	180.50	5.41
RT-DETR	89.43	82.17	90.06	63.31	83.38	12.37
RetinaNet	85.37	68.43	78.73	40.07	210.09	9.13
YOLOv5-l	94.51	91.21	95.40	81.39	109.3	8.40
YOLOv8-l	93.58	89.57	94.27	79.55	164.8	7.46
YOLOv9-c	95.10	89.85	94.64	79.99	102.3	12.29
YOLOv10-l	94.99	85.57	93.33	78.69	120.0	9.21
YOLOv12-l	95.81	92.79	96.17	81.03	95.10	10.05
YOLOv11-l	94.40	92.26	96.08	81.61	86.60	11.24
**PLD-YOLO**	**97.75**	**96.77**	**98.58**	**84.08**	**115.8**	**11.44**

Unless otherwise specified, the experimental results presented in this study were obtained using a random seed of 42.

Bold values indicate the results of the proposed PLD-YOLO model.

Compared with YOLO series models such as YOLOv9-c, YOLOv8-l and YOLOv5-l, PLD-YOLO improves the mAP@50–95 by 4.09%, 4.53% and 2.69%, respectively. This indicates that it can still maintain high localization accuracy under high IoU thresholds, especially for small object pest and disease detection tasks in complex backgrounds.

Compared with the latest YOLOv12-l, PLD-YOLO improves precision, recall, mAP@50 and mAP@50–95 by 1.94%, 3.98%, 2.41% and 3.05%, respectively. Although YOLOv12-l achieves competitive performance due to its enhanced feature representation capability, its general-purpose architecture is not specifically optimized for small-scale and fine-grained tomato disease targets.

Compared with RT-DETR of transformer structure, PLD-YOLO improves the mAP@50 and mAP@50–95 by 8.52% and 20.77%, respectively. This indicates that PLD-YOLO maintains stronger detection performance in terms of feature representation and object recall in complex backgrounds.

Compared with two-stage object detection models, PLD-YOLO improves the mAP@50 by 34.10% and 19.85% and the mAP@50–95 by 43.66% and 44.01%, respectively, compared with Faster R-CNN and RetinaNet. This indicates the limitations of traditional region proposal and anchor-based mechanisms in feature representation for small object pest and disease detection tasks.

At the same time, compared with YOLOv10-l, PLD-YOLO improves the recall by 11.20%, indicating that the model can effectively reduce missed detections in dense objects and occluded scenarios.

**Figure 5:**
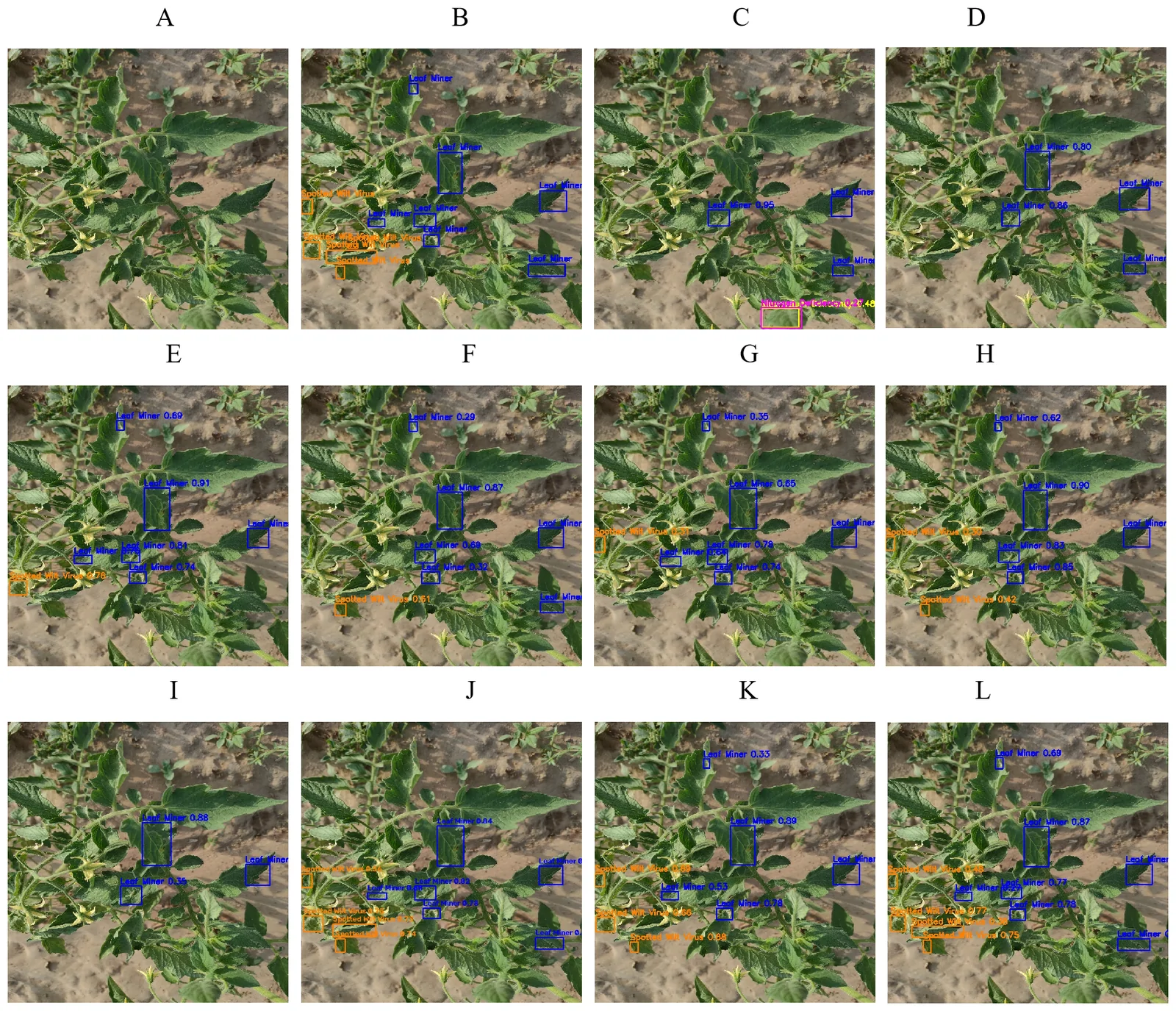
Visualization of Comparative Results of Different Models. **(A)** Original image; **(B)** ground truth; **(C)** faster R-CNN; **(D)** RetinaNet; **(E)** RT-DETR; **(F)** YOLOv5-l; **(G)** YOLOv8-l; **(H)** YOLOv9-c; **(I)** YOLOv10-l; **(J)** YOLOv12-l; **(K)** YOLOv11-l; **(L)**PLD-YOLO.

To sum up, the detection results in **Table 1** demonstrate the effectiveness of the proposed model and its strong performance in small object pest and disease detection. The visualization results of the compared models are shown in **Figure 5**. It can be observed that most models fail to fully extract feature information from images, resulting in missed detections and false detections. In particular, for pest and disease objects with small morphological differences or occlusions, the detection performance significantly degrades. Even the best-performing YOLOv11-l model still suffers from missed detections in complex natural environments. In contrast, the proposed PLD-YOLO model can accurately identify pest and disease objects, with more stable bounding boxes, and shows better performance in complex natural environments for tomato pest and disease detection.

In addition, the GFLOPs and FPS of PLD-YOLO are 115.8 G and 11.44 images/s, respectively. Compared with YOLOv11-l, the GFLOPs of PLD-YOLO increased from 86.6 G to 115.8 G, mainly because the introduced ${\text{P}}_{2}$ high-resolution detection branch and LCFE module added additional feature extraction and fusion computations. However, the inference speed of PLD-YOLO remained comparable to that of YOLOv11-l, indicating that the proposed modules enhance feature representation capability and detection accuracy without introducing significant inference latency.

Furthermore, PLD-YOLO achieved higher inference speed than Faster-RCNN, RetinaNet, YOLOv5-l, YOLOv8-l, YOLOv10-l and YOLOv12-l. This demonstrates that PLD-YOLO achieves a favorable balance between computational efficiency and detection performance while maintaining its accuracy advantages. In summary, the PLD-YOLO model proposed in this paper demonstrates excellent overall performance in tomato pest and disease detection, particularly in complex backgrounds and small object detection.

To investigate the effect of random parameter initialization on model performance during deep learning training, five independent training experiments were conducted under identical training settings using five commonly used random seeds (0, 1, 42, 1024, and 3407). These experiments were performed to evaluate the reproducibility and stability of the proposed PLD-YOLO model. The best performing model from each experiment was selected and evaluated on the test set, and the corresponding Precision, Recall, mAP@50, and mAP@50–95 values were recorded. **Table 2** presents the performance of PLD-YOLO under different random seeds, while illustrates the mean values and standard deviations (std) of each evaluation metric across the five experiments.

**Table 2:** Performance of PLD-YOLO under different random seeds.

Seed	Precision/%	Recall/%	mAP@50/%	mAP@50-95/%
0	97.08	96.03	98.31	83.96
1	97.51	96.74	98.52	83.48
**42**	**97.75**	**96.77**	**98.58**	**84.08**
1024	96.89	94.46	97.68	83.18
3407	96.54	96.43	98.17	83.88

The bold values indicate that unless otherwise specified, the experimental results presented in this study were obtained using a random seed of 42.

**Figure 6:**
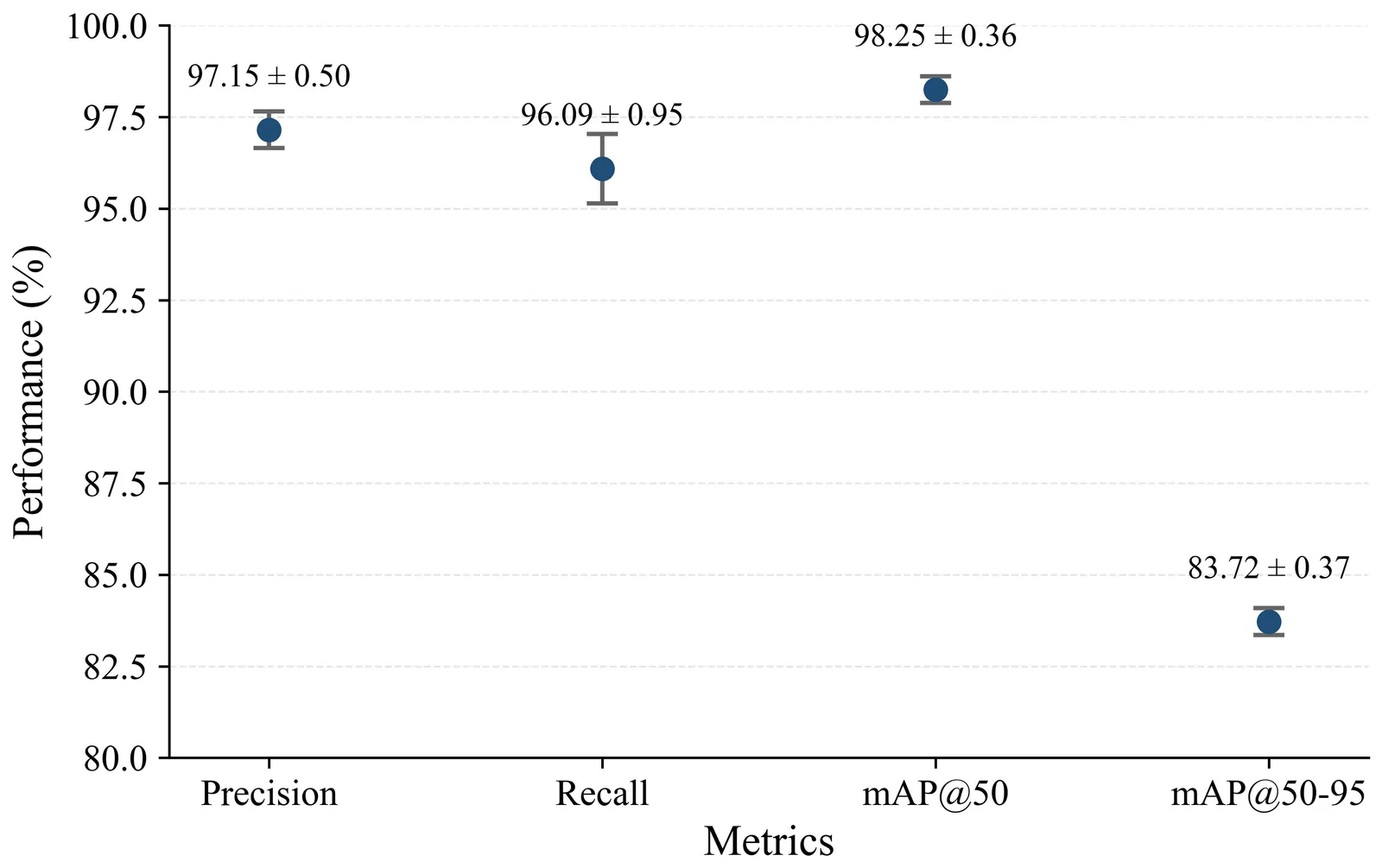
Stability analysis of PLD-YOLO under different random seeds. The points represent the mean values, and the error bars indicate the standard deviations (mean ± std) obtained from five independent experiments.

As shown in **Table 2**, the PLD-YOLO model exhibited stable detection performance across different random seeds, with only slight variations among the experimental results. As shown in **Figure 6**, the standard deviations of Precision, Recall, mAP@50, and mAP@50–95 were 0.50%, 0.95%, 0.36%, and 0.37%, respectively. All evaluation metrics exhibited standard deviations below 1%. These results indicate that random parameter initialization has a limited impact on the performance of PLD-YOLO, enabling the model to maintain stable detection performance under different random seeds.

### 4.3 Results under different scenarios

**Figure 7:**
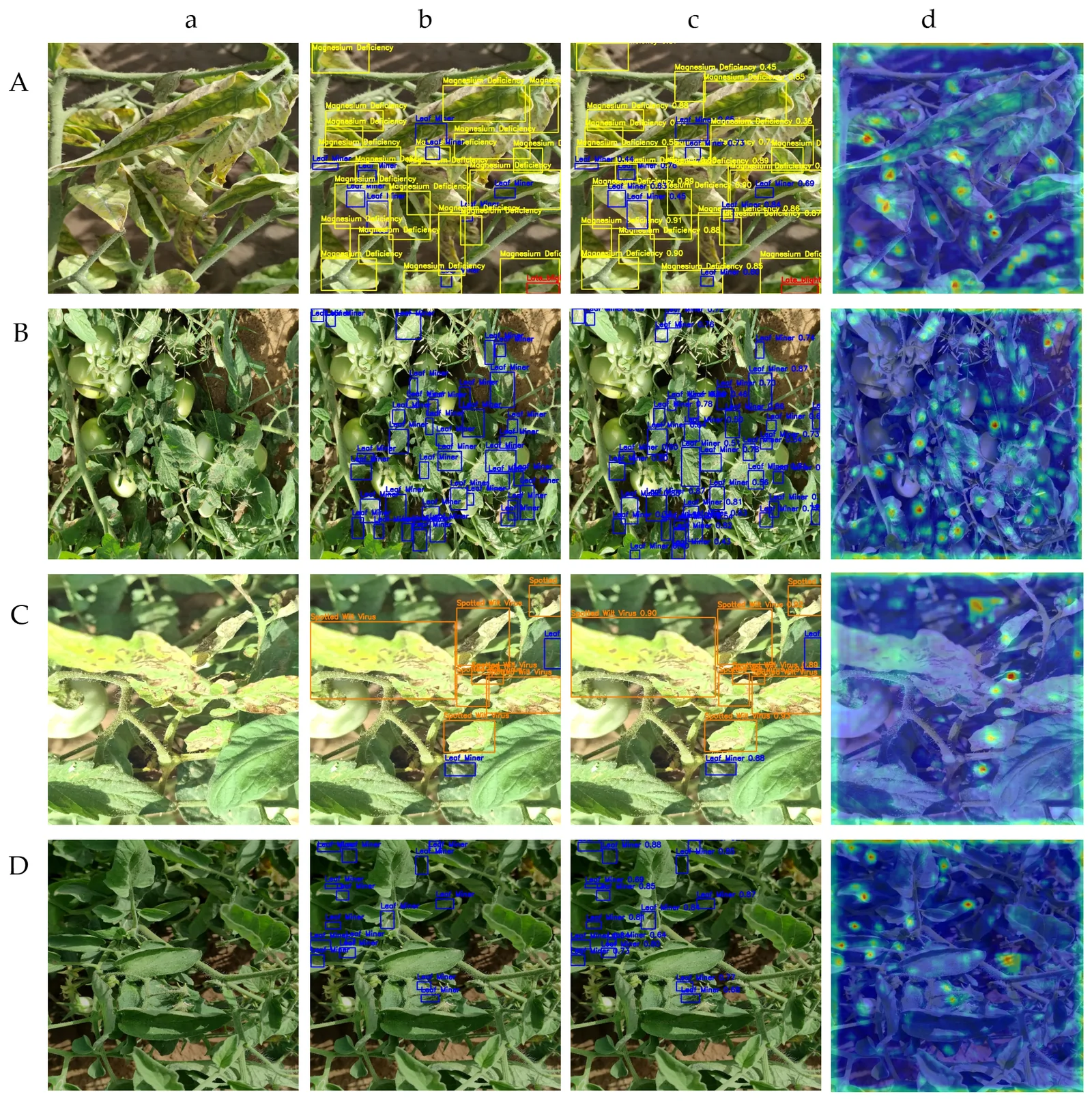
Detection Results Under Different Scenarios. **(A)** Overlap; **(B)** Density; **(C)** High light; **(D)** Low light; **(a)** Original image; **(b)** Ground truth; **(c)** Prediction results; **(d)** Heatmap.

To evaluate the detection performance of the proposed PLD-YOLO model under different natural environments, images of pest and disease objects in density, overlap, and varying illumination scenarios (including high light and low light conditions) were selected from the test set for visualization. The results in **Figure 7** show that PLD-YOLO achieves good performance in density and overlap scenarios. However, under illumination interference, especially high light conditions, edge and texture information are degraded, which may lead to local false detections. **Figure 7** further presents the results of image, Ground truth, prediction results, and heatmap, demonstrating the effectiveness of PLD-YOLO for small object pest and disease detection.

### 4.4 Ablation study

In this paper, the test set, separated from the dataset, is used to evaluate the performance of the model. The effects of the network and modules used in the improvement are analyzed. The experiments show that the best performance is achieved when all three modules are used simultaneously. It can be clearly seen from **Table 3** that the performance of the model gradually improves as the P_2_ branch, LCFE module, and DSOR-Loss are added step by step.

**Table 3:** Ablation study.

Method	P_2_	LCFE	DSOR-Loss	Precision/%	Recall/%	mAP@50/%	mAP@50-95/%	Parameters/M
YOLOV11l	×	×	×	94.40	92.26	96.08	81.61	25.29
YOLOV11l-P_2_	√	×	×	96.61	94.37	96.44	81.57	26.22
YOLOV11l-LCFE	×	√	×	95.87	94.32	97.11	81.67	25.91
YOLOV11l- DSOR-Loss	×	×	√	94.48	94.78	97.19	80.58	25.31
YOLOV11l-P_2_- LCFE	√	√	×	97.33	94.78	97.39	81.95	26.72
YOLOV11l-P_2_- DSOR-Loss	√	×	√	97.07	94.90	97.39	81.31	26.34
YOLOV11l-LCFE- DSOR-Loss	×	√	√	96.32	94.84	97.03	81.15	26.32
PLD-YOLO	√	√	√	97.75	96.77	98.58	84.08	27.43

A check mark (√) indicates that the module is used; a cross (×) indicates that it is not used.

As shown in **Table 3**, when the three modules are used individually, each module exhibits its own strengths. The introduction of the P_2_ branch module increases model precision and recall by 2.21% and 2.11%, respectively. The mAP@50 improves by 0.36%, while mAP@50–95 slightly decreases by 0.04%. When only the LCFE module is introduced, the model’s precision and recall increase by 1.47% and 2.06%, respectively. The mAP@50 and mAP@50–95 increase by 1.03% and 0.06%, respectively. When only the DSOR-Loss is introduced, the model’s precision increases slightly, recall increases by 2.52%, mAP@50 increases by 1.11%, and mAP@50–95 decreases by 1.03%.

When the modules are combined, the complementary effects of the three are further confirmed. When the P_2_ and LCFE modules are used together, the model’s precision, recall, mAP@50, and mAP@50–95 improve compared with using a single module. Compared with using the LCFE module alone, precision, recall, mAP@50, and mAP@50–95 increase by 1.46%, 0.46%, 0.28%, and 0.28%, respectively. Compared with adding only the P_2_ branch, precision, recall, and mAP@50 increase by 0.72%, 0.41%, and 0.95%, respectively. When the P_2_ branch and DSOR-Loss are used together, compared with using DSOR-Loss alone, all metrics improve to some extent: precision, recall, mAP@50, and mAP@50–95 increase by 2.59%, 0.12%, 0.20%, and 0.73%, respectively. However, compared with using the P_2_ branch alone, mAP@50–95 decreases by 0.26%. When the LCFE module and DSOR-Loss are used together, mAP@50–95 decreases by 0.52% compared with using the LCFE module alone, while precision and recall increase by 0.45% and 0.52%, respectively.

When all three modules are used together, the overall performance of the model is the best. Precision, recall, mAP@50, and mAP@50–95 reach 97.75%, 96.77%, 98.58%, and 84.08%, respectively. Compared with YOLOv11-l, mAP@50–95 increases by 2.47%, showing a significant improvement.

In summary, the modules introduced in this study play important roles in tomato leaf pest and disease detection, and achieve better performance when working collaboratively.

### 4.5 Validation experiments

This study constructs a subset of tomato leaf pest and disease images from the PlantDoc dataset and evaluates the proposed model on this subset. Compared with the Tomato-Village dataset, this subset exhibits differences in imaging conditions, background complexity, and illumination changes. Therefore, it can be used to further evaluate the stability of the model. During the testing process, the trained models were directly applied to the selected images without additional fine-tuning to objectively evaluate their adaptability to different data sources. Furthermore, to ensure a fair comparison of cross-dataset detection performance, all compared models were evaluated under identical training settings. The test results are shown in **Table 4**.

**Table 4:** Performance comparison of different object detection models on the PlantDoc dataset.

Network model	Precision/%	Recall/%	mAP@50/%	mAP@50-95/%
Faster R-CNN	58.14	54.07	49.81	39.28
RT-DETR	72.3	67.33	68.84	58.26
RetinaNet	62.48	52.46	57.36	43.18
YOLOv5-l	74.53	70.18	68.73	61.77
YOLOv8-l	76.96	71.45	69.82	63.18
YOLOv9-c	76.69	71.37	69.87	62.71
YOLOv10-l	75.40	69.32	68.15	61.27
YOLOv12-l	77.41	73.18	71.02	64.07
YOLOv11-l	77.13	72.86	70.76	63.95
**PLD-YOLO**	**78.04**	**74.4**	**71.75**	**65.84**

Bold values indicate the results of the proposed PLD-YOLO model.

Due to the differences between the PlantDoc and Tomato-Village datasets in data sources, imaging conditions, and background complexity, all compared models showed different degrees of performance degradation on the PlantDoc dataset. As shown in **Table 4**, PLD-YOLO still achieved the best detection performance among all compared models, with a Precision, Recall, mAP@50, and mAP@50–95 of 78.04%, 74.40%, 71.75%, and 65.84%, respectively. Compared with YOLOv11-l, PLD-YOLO still achieved higher detection accuracy on the external dataset, indicating that PLD-YOLO has better adaptability to data from different sources compared with existing models.

**Figure 8:**
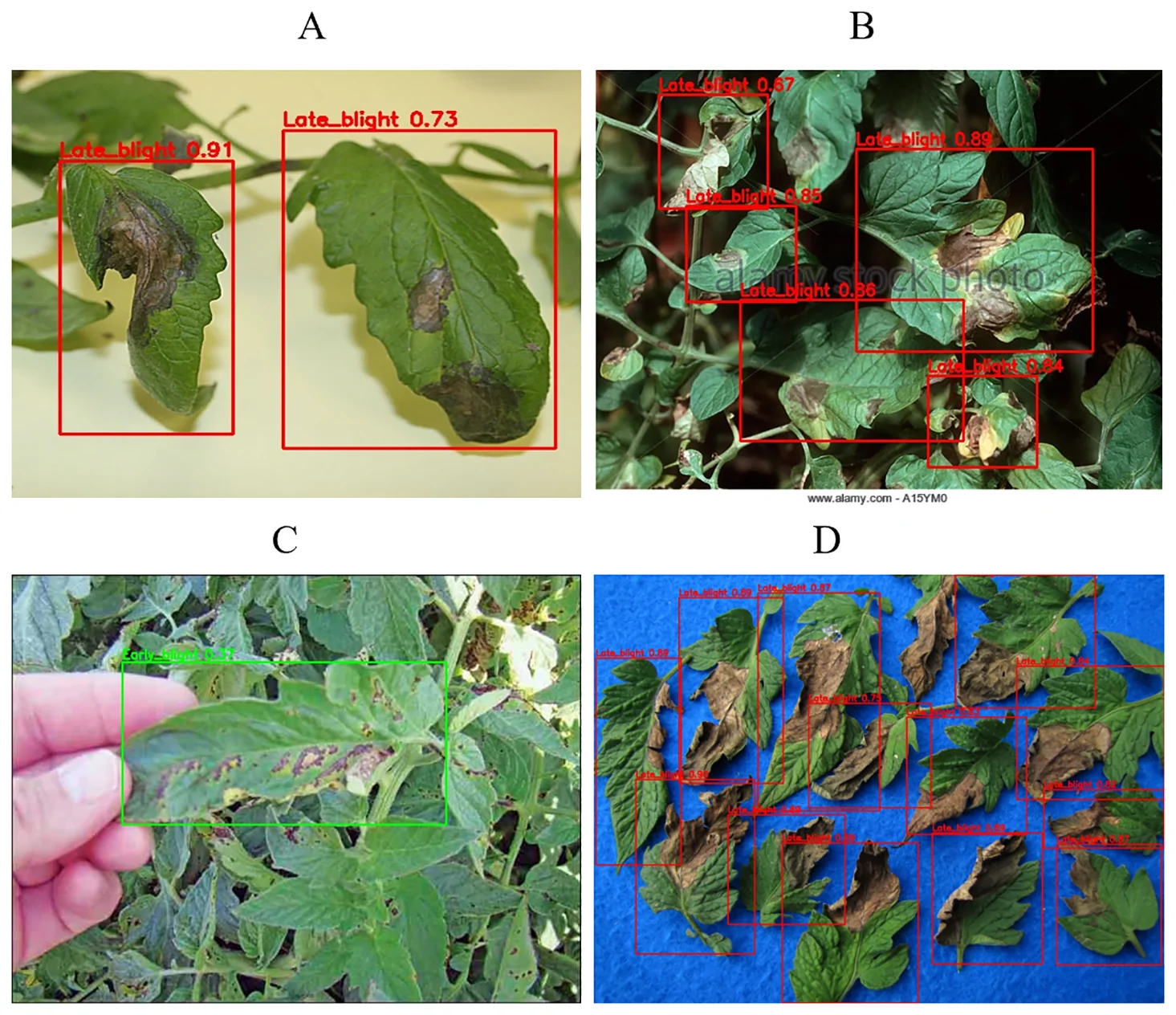
Visualization of PLD-YOLO detection results under different scenarios on the PlantDoc subset. **(A)** Simple scenario; **(B)** complex background scenario; **(C)** small object scenario; **(D)** dense scenario.

**Figure 8** shows selected detection results of PLD-YOLO on the PlantDoc subset. The visualization results show that the model can accurately detect different categories of pests and diseases in scenarios with simple backgrounds. In complex natural environments or class-overlapping scenarios, the model can still detect most pests and diseases. However, some small object lesions are missed. In densely distributed leaf scenarios, some missed detections are observed. In addition, different illumination conditions also affect the detection performance. Under uniform illumination, the model can accurately identify disease regions. However, under varying illumination and local shadow conditions, the edges and texture features of lesions are affected, resulting in slightly inaccurate localization of a small number of targets. In terms of disease types, the model can accurately identify diseases such as Late blight and Early blight. However, a small number of missed detections are observed for lesions with small sizes or indistinct boundaries. The quantitative metrics and visualization results show that PLD-YOLO achieves good object localization and category recognition performance across different scenarios. This demonstrates the adaptability of the model and its potential for application.

## 5 Discussion

In this study, a PLD-YOLO model for tomato leaf pest and disease detection in complex natural environments was proposed, focusing on improving the detection performance of small and medium-sized objects. The model achieves overall performance improvements on the Tomato-Village dataset, demonstrating stronger adaptability and robustness in complex natural environments.

The performance improvement is mainly due to the synergistic effects of the different modules. The introduced P_2_ high-resolution branch enhances the feature representation of small objects by preserving shallow feature information, providing advantages for detecting dense and small-scale pest and disease objects. However, due to insufficient high-level semantic information, directly using shallow features may lead to slight localization deviations under high IoU thresholds. The LCFE module enhances feature representation by introducing local contextual information, enabling more accurate object-background discrimination in complex environments and improving discriminative capability. However, its effect is more significant on classification and object existence prediction, while its impact on bounding box regression remains limited, resulting in constrained improvements under high IoU thresholds. The DSOR-Loss improves the model’s sensitivity to objects by adaptively adjusting sample weights, enabling more objects to be detected. However, by assigning higher weights to small objects, it slightly affects the stability of bounding box regression. This leads to limited performance improvement under high IoU thresholds.

When all three modules are integrated, the model achieves the best overall performance. Compared with the baseline model, mAP@50–95 is significantly improved by 2.47%, highlighting the complementary roles of the different modules. The fine-grained spatial information provided by the P_2_ branch forms the basis for small object detection. The LCFE module filters and enhances features, partially suppressing interference from complex backgrounds. The DSOR-Loss strengthens small object detection from an optimization perspective, guiding the model to focus more on hard-to-detect samples during training. Under the cooperation of these modules, the model not only improves detection completeness but also reduces false detections in complex scenarios. This alleviates the limitations of individual modules under high IoU thresholds and further enhances overall detection performance.

Deep learning models are usually affected by factors such as random parameter initialization and data perturbations during the training process. The random seed experiments show that PLD-YOLO maintains relatively small performance fluctuations across five different random seeds, demonstrating stable detection performance. This indicates that the detection performance improvement of the model is not caused by random variations during training, but mainly results from the effective feature representation capability of the proposed network architecture and optimization strategy for pest and disease features. PLD-YOLO exhibits small fluctuations in Precision, mAP@50, and mAP@50-95, indicating high stability in overall detection performance. Although Recall shows relatively larger fluctuations, its standard deviation remains below 1%. This may be because the detection results of some challenging samples, such as small object lesions and regions with indistinct features, are more sensitive to randomness during training. Overall, the random seed experiments further validate the training stability and reproducibility of PLD-YOLO in tomato leaf pest and disease detection.

To evaluate the model’s adaptability under different environmental conditions, this study conducted external validation experiments using tomato pest and disease images selected from the PlantDoc dataset. Compared with the Tomato-Village dataset, all models exhibited different degrees of performance degradation on the PlantDoc dataset. This may be attributed to the differences in data sources, image acquisition conditions, background complexity, and illumination conditions between the two datasets. The Tomato-Village dataset was collected from real tomato cultivation environments, whereas the PlantDoc dataset consists of multi-source images and publicly available images from the Internet. These data distribution differences increase the difficulty of extracting features from pests and diseases and discriminating their categories. However, PLD-YOLO still achieved the best overall detection performance among all compared models. This may be because PLD-YOLO effectively utilizes fine-grained information and local context features to extract more discriminative features of pests and diseases. These results indicate that PLD-YOLO has good adaptability for tomato leaf pest and disease detection under different environmental conditions.

Although PLD-YOLO has achieved significant performance improvements in tomato leaf pest and disease detection, the model still has certain limitations. First, the proposed method is mainly optimized for small object detection and small- and medium-sized object detection. By enhancing fine-grained feature representation, it improves the detection ability for small lesions. However, the improvement for large-scale object detection remains relatively limited. Second, although PLD-YOLO exhibits good adaptability under different environmental conditions, it may still be affected by factors such as background interference, object occlusion, and appearance variations of pests and diseases in complex natural environments, resulting in missed and false detections for some challenging samples. In addition, PLD-YOLO is based on YOLOv11-l, which has a relatively large number of parameters. In practical agricultural applications requiring lightweight deployment, the model still imposes considerable computational demands, making it less suitable for resource-constrained devices. Furthermore, domain adaptation and domain generalization strategies will be explored in future work to reduce the distribution gap between training and deployment environments. We also plan to construct a field-collected tomato pest and disease dataset from diverse cultivation environments to further evaluate the adaptability of PLD-YOLO and improve its performance under practical agricultural scenarios.

## 6 Conclusion

This paper presents an improved PLD-YOLO model for small object detection of tomato leaf pest and disease in complex natural environments. The proposed method effectively addresses the challenges of feature loss in small objects, dense object distribution, and strong background interference, enabling accurate detection of pest and disease in complex natural environments.

The experimental results show that the PLD-YOLO model outperforms other comparative models in key metrics, including precision, recall, mAP@50, and mAP@50-95. In particular, the improvement in mAP@50–95 is more pronounced, further demonstrating the model’s superior performance under more stringent evaluation conditions. Visualization results indicate that the PLD-YOLO model effectively reduces missed and false detections in dense object regions and complex backgrounds. Furthermore, random seed experiments demonstrate that PLD-YOLO maintains stable detection performance under different initialization conditions, validating its training stability and reproducibility. External validation experiments on the PlantDoc dataset further demonstrate the adaptability of the model under different data distributions and environmental conditions. PLD-YOLO provides a potential technical solution for intelligent plant protection in complex agricultural environments.

The backbone network based on YOLOv11-l has a large number of parameters. In future work, we will explore lightweight network architectures to reduce computational complexity and improve the real-time performance of the model on mobile devices, thereby better supporting the development of smart agriculture. In addition, more effective feature enhancement, adaptive learning, and domain adaptation strategies will be explored to further improve detection performance and adaptability under diverse agricultural scenarios.

## Data Availability

The original contributions presented in the study are included in the article/**Supplementary Material**. Further inquiries can be directed to the corresponding author.
